# Supplementary material for: Multi‐dataset identification of innovative feature genes and molecular mechanisms in keratoconus
Source: J Cell Mol Med. 2024 Sep 19;28(18):e70079. doi: 10.1111/jcmm.70079 (PMC11412914; doi:10.1111/jcmm.70079)
Supplement: Supplementary file 2 — Material S2. [file JCMM-28-e70079-s002.zip › 7.GSEA analysis/5.RNF217-AS1/KEGG_ASTHMA.html]

Details for gene set KEGG\_ASTHMA[GSEA]

|  || Dataset | RNF217-AS1.group.cls#h\_versus\_l |
| Phenotype | group.cls#h\_versus\_l |
| Upregulated in class | l |
| GeneSet | KEGG\_ASTHMA |
| Enrichment Score (ES) | -0.5836968 |
| Normalized Enrichment Score (NES) | -1.2990383 |
| Nominal p-value | 0.17281553 |
| FDR q-value | 0.4026398 |
| FWER p-Value | 0.954 |
Table: GSEA Results Summary

  

Fig 1: Enrichment plot: KEGG\_ASTHMA      
 Profile of the Running ES Score & Positions of GeneSet Members on the Rank Ordered List

  

| SYMBOL | TITLE | RANK IN GENE LIST | RANK METRIC SCORE | RUNNING ES | CORE ENRICHMENT || 1 | IL9 | na | 1603 | 0.382 | 0.0187 | No |
| 2 | IL3 | na | 4732 | 0.226 | -0.0329 | No |
| 3 | IL13 | na | 8644 | 0.113 | -0.1264 | No |
| 4 | CCL11 | na | 10610 | 0.076 | -0.1701 | No |
| 5 | HLA-DOA | na | 11639 | 0.061 | -0.1894 | No |
| 6 | TNF | na | 13583 | 0.035 | -0.2395 | No |
| 7 | HLA-DMB | na | 13802 | 0.033 | -0.2402 | No |
| 8 | HLA-DMA | na | 14233 | 0.028 | -0.2478 | No |
| 9 | HLA-DRB5 | na | 19471 | -0.025 | -0.3945 | No |
| 10 | HLA-DRB1 | na | 23407 | -0.067 | -0.4965 | No |
| 11 | IL5 | na | 23582 | -0.070 | -0.4897 | No |
| 12 | CD40 | na | 23601 | -0.070 | -0.4784 | No |
| 13 | HLA-DRB3 | na | 23619 | -0.070 | -0.4670 | No |
| 14 | HLA-DOB | na | 26792 | -0.122 | -0.5377 | No |
| 15 | FCER1G | na | 27239 | -0.131 | -0.5283 | No |
| 16 | IL10 | na | 28464 | -0.161 | -0.5363 | No |
| 17 | CD40LG | na | 30110 | -0.210 | -0.5480 | Yes |
| 18 | HLA-DRB4 | na | 30111 | -0.210 | -0.5123 | Yes |
| 19 | PRG2 | na | 30129 | -0.211 | -0.4770 | Yes |
| 20 | HLA-DPA1 | na | 30869 | -0.237 | -0.4581 | Yes |
| 21 | MS4A2 | na | 31176 | -0.249 | -0.4246 | Yes |
| 22 | HLA-DQB1 | na | 31211 | -0.250 | -0.3830 | Yes |
| 23 | HLA-DRA | na | 31261 | -0.252 | -0.3415 | Yes |
| 24 | HLA-DQA1 | na | 32266 | -0.307 | -0.3182 | Yes |
| 25 | EPX | na | 32540 | -0.327 | -0.2705 | Yes |
| 26 | IL4 | na | 33228 | -0.378 | -0.2260 | Yes |
| 27 | HLA-DQA2 | na | 33332 | -0.388 | -0.1630 | Yes |
| 28 | HLA-DPB1 | na | 33623 | -0.423 | -0.0995 | Yes |
| 29 | FCER1A | na | 34631 | -0.773 | 0.0028 | Yes |
Table: GSEA details [plain text format]

  

Fig 2: KEGG\_ASTHMA      
 Blue-Pink O' Gram in the Space of the Analyzed GeneSet

  

Fig 3: KEGG\_ASTHMA: Random ES distribution      
 Gene set null distribution of ES for **KEGG\_ASTHMA**
